# Advances in Therapeutic Vaccines Against HPV: A Review of Human Clinical Trials

**DOI:** 10.3390/curroncol32110600

**Published:** 2025-10-25

**Authors:** Elena Martín, Gabriel Reina, Silvia Carlos

**Affiliations:** 1 School of Pharmacy and Nutrition, Universidad de Navarra, 31008 Pamplona, Spain; emartinlope@alumni.unav.es; 2 Microbiology Department, Clínica Universidad de Navarra, 31008 Pamplona, Spain; 3 IdiSNA, Navarra Institute for Health Research, 31008 Pamplona, Spain; scarlos@unav.es; 4 Department of Preventive Medicine and Public Health, Universidad de Navarra, 31008 Pamplona, Spain

**Keywords:** human papillomavirus (HPV), cervical cancer, therapeutic vaccines, viral clearance, DNA vaccines, clinical trials, low- and middle-income countries (LMICs)

## Abstract

**Simple Summary:**

Cervical cancer continues to be one of the most common and deadly cancers among women, especially in countries with limited healthcare resources. It is caused by high-risk types of human papillomavirus (HPV). While existing vaccines can prevent infection, they cannot treat women who already have HPV-related lesions. This study reviews current research on therapeutic HPV vaccines, which aim to help the body eliminate the virus and stop the progression to cancer. Several types of vaccines have shown promise in early clinical trials, particularly DNA-based ones. However, more research is needed before these vaccines can become widely available. Understanding how these vaccines work and improving their effectiveness could make a big difference in global efforts to reduce cervical cancer, especially where preventive options are limited.

**Abstract:**

Cervical cancer remains a major public health concern, particularly in low- and middle-income countries (LMICs) where access to preventive measures is limited. Persistent infection with high-risk human papillomavirus (HPV) types, mainly HPV16 and HPV18, is the key cause of cervical cancer. While prophylactic HPV vaccines effectively prevent new infections, they offer no therapeutic benefit for individuals with established lesions. This review evaluates the clinical evidence on therapeutic HPV vaccines, focusing on their ability to promote viral clearance. A bibliographic search was conducted in PubMed, selecting human studies reporting outcomes on HPV clearance. Seventeen clinical trials were identified, including DNA-based (VGX-3100, GX-188E), viral-vector (MVA E2, TG4001), peptide-based (Pepcan), and bacterial-vector (GLT 001) vaccines. Among them, DNA-based vaccines, particularly VGX-3100, showed the most consistent results, whereas several protein- or vector-based approaches demonstrated variable outcomes. Early therapeutic HPV vaccine trials faced setbacks due to limited efficacy, delivery approaches, and study design challenges, preventing progression to late-phase development. Recent DNA-based candidates, however, are advancing through phase II/III trials. While none have yet to be approved for commercial use, these vaccines elicit virus-specific T-cell responses and can induce regression of precancerous lesions, offering a promising addition to prophylactic vaccination and screening. Variability in study designs and endpoints underlines the need for standardized protocols and further phase III trials. Overall, therapeutic HPV vaccines represent a rapidly advancing field with the potential to complement prophylactic vaccination and screening, thereby strengthening global cervical cancer control efforts, particularly in LMICs.

## 1. Introduction

Worldwide, cervical cancer (CC) ranks as the fourth most prevalent cancer among women, with approximately 660,000 new cases reported in 2022. In that year, around 94% of the 350,000 deaths caused by CC took place in low- and middle-income countries (LMICs). The highest incidence and mortality rates of CC are found in sub-Saharan Africa (SSA), Central America, and Southeast Asia [1]. In particular, SSA has the highest rate of human papillomavirus (HPV) infection (CC causative agent) globally (7.2 million cases in a population of 58 million) [2]. These numbers are aggravated by the fact that around 13 million women in SSA are estimated to be infected with HIV [3], a key risk factor that promotes the persistence of HPV infection and CC development [2].

The infection with oncogenic HPV types is the necessary cause of CC, although there are other exogenous and endogenous factors that jointly affect the likelihood of advancing from cervical HPV infection to CC, such as the presence of other sexually transmitted infections, smoking, use of hormonal contraceptives, or high parity [4].

Considering their connection to cervical preneoplastic and neoplastic lesions, HPV can be classified into low- and high-oncogenic-risk types. Low-risk HPV (LR-HPV) include types 6, 11, 42, 43, 44, 54, 61, 70, 72, and 81. High-risk HPV (HR-HPV) variants consist of types 16, 18, 31, 33, 35, 39, 45, 51, 52, 56, 58, 59, 68, 73, and 82. HPV types 6 and 11 are the causal agents in nearly all genital warts and in a minor percentage of temporary cervical lesions (cervical intraepithelial neoplasia, CIN1). Viral types 16 and 18 account for 70% of cervical cancers and approximately 50% of precancerous lesions (CIN2/3) [4,5].

HPV is a small circular double-stranded DNA virus that has a viral capsid organized in a naked icosahedral structure. The genome encodes several proteins, including L1 and L2 (structural proteins which conform the viral capsid); E6 and E7 proteins, which promote viral replication and oncogenesis; and E2, responsible for viral transcription [6]. When E6 and E7 are expressed at controlled levels, their main role is to regulate the host cell cycle, supporting the maintenance of the viral genome. Only when the expression of these proteins becomes unregulated, can they be considered oncogenes. These oncoproteins tightly bind to and inactivate key tumor suppressors, such as p53 and retinoblastoma protein (pRb). This disruption allows cells to evade normal cell cycle control, promotes uncontrolled cell proliferation, and facilitates mutations that drive cancer development. Additionally, disrupting p53 and pRb impairs DNA repair mechanisms, increasing genomic instability and creating conditions that favor viral DNA integration into the host genome, further accelerating oncogenic transformation through unchecked oncoprotein activity [5,7,8]. L1 proteins are the basis of prophylactic vaccines through the induction of L1-specific neutralizing antibodies since they contain HPV type-specific virus-like particles obtained from L1 [9], while E6, E7, and E2 are common proteins used for therapeutic vaccines.

HPV infection alone is not a sufficient cause of CC. Other host systems and behavioral and environmental co-factors determine cancer development. Some well-known co-factors are HIV infection, the presence of other sexually transmitted infections (STI) (e.g., *Chlamydia trachomatis* or *Trichomonas vaginalis*), smoking, use of hormonal contraceptives, and high parity [10]. HPV is primarily transmitted through close skin-to-skin contact during sex (vaginal, anal, or oral sex). An individual with HPV can transmit the infection to another person even if they show no signs or symptoms [11].

Most HPV infections are cleared by the immune system, but if they persist (about 10–20% persist asymptomatically) and are left untreated, they progress to precancerous cells and then to cancerous cells [12]. Due to persistent HPV infection, cervical epithelium cells suffer dysplasia, and these precursor lesions are named cervical intraepithelial neoplasia (CIN). The magnitude of dysplasia represents severity; therefore, they are classified as CIN1, 2, or 3 [13]. They can also be classified by a two-stage system, High-grade Squamous Intraepithelial Lesion (HSIL) and Low-grade Squamous Intraepithelial Lesion (LSIL) [14].

Since CC is a global public health problem, in 2020, the World Health Organization (WHO) developed a Global Strategy for the Elimination of Cervical Cancer. This strategy proposed the “90-70-90 targets” focused on three objectives: (1) full vaccination with the HPV vaccine of 90% of girls by age 15, (2) screening of 70% of women by the age of 35 and again at 45, and (3) treatment of 90% of women with precancerous lesions and management of invasive cervical cancer [15]. Every country should achieve the 90-70-90 goals by 2030 to facilitate eradicating cervical cancer globally.

In this sense, **primary prevention** strategies are essential to reduce the incidence of HPV infection and development of CC. Since CC-associated HPV types are sexually transmitted, the first option to prevent the disease is having an effective sexual education program, as risk behaviors like early sex, multiple sexual partners, or incorrect/inconsistent use of condoms increase the risk for STIs. HPV can be transmitted despite the use of condoms through skin-to-skin contact in areas not covered by condoms [16,17,18].

Prophylactic vaccines were introduced in 2006 with Gardasil 4, which offered immunization against HPV types 6, 11, 16 and 18. New prophylactic vaccines have been approved, such as a bivalent vaccine against types 16 and 18 or a more complete nonavalent vaccine called Gardasil 9 that offers protection from types 31, 33, 45, 52, and 58 in addition to 6, 11, 16, and 18 [19].

Less than 30% of LMICs had introduced prophylactic HPV vaccines in 2020 in comparison to high-income countries [20]. SSA countries are especially delayed in reaching the 90% vaccination goal proposed by the WHO. Some barriers to successfully implementing HPV vaccination are scarcity of resources, lack of information concerning vaccination services, limited knowledge of HPV and CC, misinformation about vaccine safety, and stigma around STIs [21].

The aim of **secondary prevention** is the early identification of the disease. This can be achieved through the Papanicolaou (Pap) cytological test or, as the WHO recommends today, by a cervicovaginal HPV test that checks for the presence of oncogenic types. Screening is recommended at least every 5 years for women between ages 30 and 65 or every three years for some high-risk populations, such as women with HIV [22]. If an abnormal cervical cytology or a positive HPV test is obtained, the next step is to perform a colposcopy and biopsy to determine further treatment [23].

Finally, **tertiary prevention** of cervical cancer focuses on managing the disease after diagnosis to minimize its impact and prevent recurrence or complications. There are different approaches depending on the progression of the disease. Patients newly diagnosed with HPV infection might benefit from products such as Papilocare^®^ that may clear infection and regress LSIL [24,25]. Papilocare^®^ is composed of seven key ingredients including *Coriolus versicolor* extract (a fungus commonly used in traditional Chinese medicine) and Bioecolia^®^, a prebiotic that supports the growth of beneficial bacteria like *Lactobacillus crispatus*. The formula also features hyaluronic acid, beta-glucan, *Centella asiatica*, neem extract, and aloe vera extract (https://pro.papilocare.com/en/papilocare-vaginal-gel/, accessed on 10 October 2025).

Current treatment strategies for LSIL and HSIL focus on eliminating abnormal HPV-infected cells and include the loop electrosurgical excision procedure (LEEP), conization, cryotherapy, or thermal ablation [26]. If the diagnosis is cervical cancer, recommended treatment includes surgery, radiation therapy, chemotherapy, targeted therapy, or immunotherapy, depending on different factors [27,28].

Advancements in therapeutic vaccine development targeting HPV could prove crucial in the progression of existing strategies for preventing and managing HPV-related cancers. Most of them are in the early phases of clinical trials and could serve as an extra resource to fill gaps in CC programs. Unlike prophylactic HPV vaccines, which are designed to prevent new infections, these vaccines aim to eliminate or treat already existing HPV infections and associated precancerous lesions [7].

There are two key **contexts** where therapeutic vaccines might prove quite useful: (1) Areas were scaling up CC screening and treatment has been challenging, to reach women who have likely not received the HPV vaccine and to lower the proportion of those who may develop or already have cervical lesions; (2) regions where screening and treatment are available, to introduce a simpler treatment method [7].

Therapeutic HPV vaccines work by delivering specific target antigens to antigen-presenting cells (APCs), such as dendritic cells (DCs). These DCs then present antigenic peptides to the immune system through major histocompatibility complexes (MHC), triggering CD8^+^ and CD4^+^ T-cell responses [29].

Researchers have explored different delivery methods—peptide- or protein- based vaccines, DC-based vaccines, nucleic acid vaccines (DNA and RNA), and vector-based vaccines—each offering distinct advantages and drawbacks [30,31]. Even nanoparticles have proven to be a useful strategy in this area because of their adaptability and ability to effectively penetrate and destroy cancer cells [32].

The study aims to review the evidence on therapeutic vaccines for cervical cancer prevention, particularly for HPV clearance. As a secondary objective, the study aims to evaluate the pipeline and timeline of the development of therapeutic HPV vaccines.

## 2. Methods

A comprehensive literature review was performed to identify human clinical trials evaluating the efficacy of therapeutic vaccines on HPV infection and lesion regression. The search strategy combined structured database searches with the cross-referencing of bibliographies from relevant reviews and clinical trial registries to ensure methodological rigor.

The PubMed and Scopus databases were searched as recent as April 2025, using a combination of MeSH terms and Boolean operators: (“cervical cancer” OR “human papillomavirus” OR “HPV”) AND (“therapeutic vaccine” OR “immunotherapy”). Only studies conducted on humans and published in English were considered.

First, we searched for all the reviews that included the terms described above, to check that this review had not been previously performed. After that, a second search was carried out adding “clearance” to the initial MeSH terms. Finally, a third search was carried out including the term “clinical trials”.

The titles and abstracts were independently screened by two reviewers, and full texts were assessed to confirm eligibility. Any disagreements were resolved by consensus. The inclusion criteria comprised studies presenting quantitative or qualitative outcomes related to HPV clearance, viral load reduction, or histologic regression. The exclusion criteria included preclinical studies, reports unrelated to HPV infection, studies without full-text availability, and trials not reporting relevant endpoints.

For each study, data were systematically extracted based on vaccine platform, antigenic targets, study design, population characteristics, number of doses, route of administration, follow-up duration, and main efficacy outcomes. Differences in control groups, lesion grade distribution, and diagnostic criteria were considered as potential sources of bias. This comprehensive approach was designed to capture and synthesize all available clinical evidence on HPV therapeutic vaccines up to April 2025.

## 3. Results

### 3.1. Studies Selection

**Figure 1:**
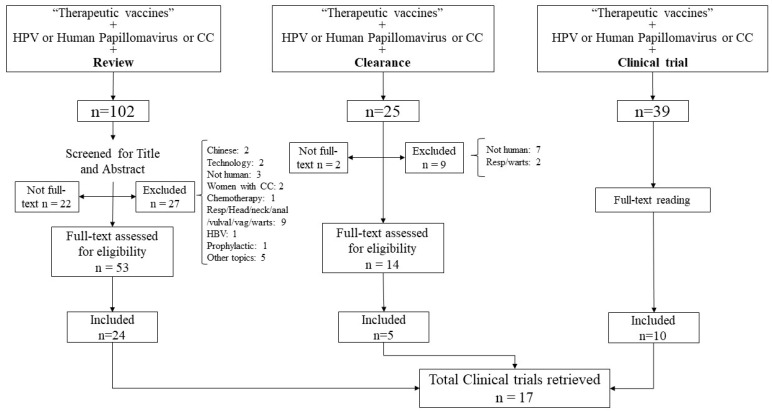
Flowchart of the studies selection.

The first search strategy to check for reviews on therapeutic vaccines and HPV/CC resulted in the identification of 102 articles, of which 24 were selected. The second search (including “clearance”) lead to 25 results, of which 3 were already included in the first search, and 5 were selected. After reading the chosen articles, 17 clinical trials were selected [33,34,35,36,37,38,39,40,41,42,43,44,45,46,47,48,49,50] (Figure 1).

### 3.2. HPV Vaccines and Clearance

Across the 17 clinical trials identified, the overall objective was to evaluate the ability of therapeutic vaccines to promote HPV clearance and histologic regression in women with CIN2/3 or related precancerous lesions. DNA-based vaccines such as VGX-3100 and GX-188E consistently showed a high immunogenicity and viral clearance rate, with up to 78% clearance among responders in phase II studies [33,34]. The use of electroporation as a delivery enhancer appeared crucial to increasing cellular uptake and gene expression of E6/E7 antigens [34]. Viral-vector vaccines, including MVA E2 and TG4001, demonstrated robust induction of HPV-specific T-cell responses and lesion regression, although progression to later-phase trials has been limited to Latin America and Europe [36,37,38,41]. Peptide- and protein-based formulations (Pep-Can, HspE7, CIGB-228) achieved partial efficacy, particularly in generating cytotoxic responses in HLA-A2-positive individuals but showed variability depending on lesion grade and adjuvant use [43,44,47,48,49]. Bacterial-based and fusion protein vaccines, such as GTL001 (ProCervix), showed promise in early trials with local application combined with immune stimulants, like imiquimod; however, the phase II trial revealed no significant difference from placebo, emphasizing the need for improved adjuvantation or dosing strategies [39]. The oral *Lactobacillus casei*-based vaccine IGMKK16E7 represented an innovative approach to mucosal delivery, though no statistical difference in viral clearance was observed, highlighting formulation challenges for mucosal immunization [50]. The follow-up duration across trials ranged from 8 weeks to 2.5 years, with most protocols administering three or four vaccine doses. Phase I/II trials generally reported viral clearance rates between 50 and 80% among vaccine responders, while control groups showed 20–40% spontaneous regression.

**Figure 2:**
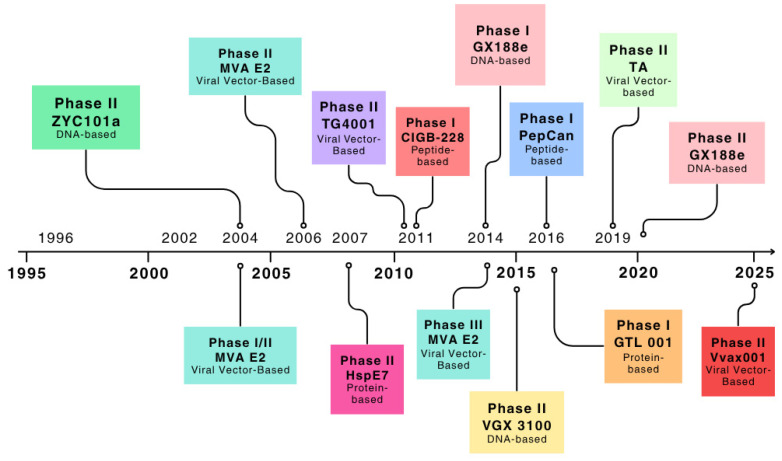
Therapeutic vaccine development pipeline [33,34,35,36,37,38,39,40,41,42,43,44,45,46,47,48,49,50].

As shown in Figure 2, research on the development of therapeutic vaccines to treat HPV goes back more than 20 years and a multitude of vaccines have gone through pre-clinical and clinical trials.

The majority of the vaccines targeted E6/E7 HPV proteins of either HPV types 16 or 18 but there was one with a different target: a viral vector vaccine, MVA E2, containing the E2 transcription-regulating protein. As of May 2025, only one vaccine had reached phase III, VGX 3100 [51]. MVA E2 had not progressed to phase III in the U.S. or Europe. Most vaccines had been tested in developed countries, as early-phase trials are often more feasible in high-resource settings, but the implementation in LMICs remains essential. It is not clear how much of the clearance is caused by natural regression because few clinical trials had a control group.

This review focused on the efficacy of the vaccines to clear HPV infection or reduce viral load. The results on clearance are summarized in Table 1.

## 4. Discussion

A strategy is clearly necessary to address the needs of the significant number of people currently living with persistent HPV infection and/or HPV-related conditions. Once HPV infection has occurred, prophylactic vaccines are no longer effective, and the only option would be to wait for the natural clearance to occur or treat the lesion as explained previously. Since recurrence of HPV infection is possible after an intervention, therapeutic vaccines play a key role in clearing up the infection and thus improving the quality of life of the patients. Furthermore, therapeutic vaccines may represent a simpler approach to successfully treat CIN lesions and eradicate the virus permanently through the stimulation of the cell-mediated immune responses that eliminate HPV-infected cells.

It seems that the most effective vaccines to date are the DNA-based vaccines, such as the VGX-3100 (2 DNA plasmids encoding E6 and E7 genes of HPV16 and HPV18) and GX-188e (DNA vaccine encoding a tissue plasminogen activator signal sequence and an Fms-like tyrosine kinase-3 ligand together with HPV16/18 E6/E7 antigens), since they are easy to produce, stable, and elicit both humoral and cellular immunological response. Antibodies against E6 and E7 are unlikely to play a major role in histopathological regression; but they may serve as biomarkers of helper T-cell activation instead of direct effectors. The main limitation presently associated with DNA vaccines is their delivery into the nuclei of host cells, necessary for the transcription of DNA plasmids into mRNA [29]. In order to enhance the potency of therapeutic HPV DNA vaccines, they can be delivered by intramuscular injection followed by electroporation (low voltage currents that create small ruptures in the cellular membrane for the plasmids to pass through) [32].

Nevertheless, vaccines which are not DNA-based have also achieved good results pertaining viral clearance. Peptide- and protein-based vaccines (e.g., PepCan, HspE7 and CIGB-228) are among the safest and most straightforward to produce. Short synthetic peptides allow for precise epitope targeting, while long peptides or recombinant proteins can induce both helper and cytotoxic T-cell activation. Nevertheless, their overall immunogenicity remains modest in the absence of potent adjuvants, and short peptides are restricted by HLA haplotypes, reducing universal applicability. Vector–based vaccines (e.g., MVA E2, TG4001, TA, GTL001 and Vvax), including attenuated viral and bacterial systems, mimic natural infection and induce strong humoral and cellular immune responses. Vectors such as modified vaccinia Ankara (MVA) have proven effective in antigen delivery and dendritic cell activation. However, pre-existing anti-vector immunity, safety concerns in immunocompromised individuals, and difficulties in repeated administration pose significant challenges [30,31].

Over the last decade, converging evidence has confirmed that therapeutic HPV vaccines can elicit potent T-cell immunity capable of mediating lesion regression and viral clearance. Meta-analyses suggest clinical response rates approaching 54% for CIN2/3 lesions when robust CD8^+^ responses are achieved, compared to 27% of pooled regression with placebo [52].

Overall, since the design, intervention protocol, vaccines doses, and route of administration in the studies were diverse, it is difficult to compare the results. In patients diagnosed with CIN2/3, the rate of viral DNA clearance, regardless of HPV type, was significantly higher in the cohorts receiving the therapeutic vaccine compared to the control group in the selected studies and a stratified trial analysis would be recommended. Nevertheless, the heterogeneity in inclusion criteria, endpoints, and immunological monitoring still limits cross-trial comparability and meta-analytic synthesis.

New mechanistic insights indicate that incomplete regression is frequently linked to the immune evasion within the cervical microenvironment, including PD-L1 up-regulation, regulatory T-cell recruitment, and local cytokine imbalance. Combination approaches pairing therapeutic vaccination with checkpoint inhibitors or TLR agonists are under active investigation and may enhance efficacy. Technological progress has also expanded beyond DNA-based and viral-vector platforms. mRNA and nanoparticle-based vaccines are now entering early-phase clinical testing, offering rapid production, improved antigen expression, and avoidance of anti-vector immunity. These innovations could help overcome the modest progress observed during the past two decades [53,54]. In addition, assessing the effector mechanisms induced by therapeutic vaccines in cervicovaginal tissue remains challenging; however, the application of innovative single-cell and multiplex immunoassays may enhance the detection and characterization of these local immune responses in future studies.

Given that LMICs are still far from achieving the WHO cervical cancer elimination targets, therapeutic vaccines could complement prophylactic approaches to reduce morbidity and mortality associated with CC. Both vaccine types may contribute to filling the identified gaps in cervical cancer prevention initiatives; consequently, there is still a need to determine a suitable niche for the therapeutic vaccines.

The WHO 2024 Preferred Product Characteristics document emphasizes harmonization of endpoints, standardized immunologic assays, and simplified dosing schedules to facilitate global implementation. If these recommendations are adopted, particularly in low- and middle-income countries where cervical cancer burden remains the highest, therapeutic vaccination could become a realistic adjunct to screening and prophylactic immunization programs [6].

Collectively, these developments reflect a transition from proof-of-concept trials toward more integrative, precision-driven vaccine strategies that may finally close the gap between experimental efficacy and widespread clinical utility.

## 5. Conclusions

Therapeutic vaccines have demonstrated immunogenicity and partial efficacy in inducing viral clearance and lesion regression, though none have reached sufficient efficacy for regulatory approval. They have shown to be effective in clearing HPV16/18 which may have a great public health impact since they are responsible for the vast majority of cervical preneoplasic and neoplasic lesions. Therefore, in areas where the prophylactic vaccine has not been implemented or access to excision treatment or screening is limited, therapeutic vaccines will be a valuable tool to reach women that have not been vaccinated to reduce the percentage who will inevitably acquire the HPV virus and could subsequently develop cancer. No vaccine has been approved for commercialization and much research is still needed to improve their efficacy and determine their role in CC treatment. Implementation concerns, such as feasibility, acceptability, reach, cost, and sustainability, need to be assessed in both high- and low-resource settings.

## Figures and Tables

**Table 1:** Data on clearance outcome for therapeutic vaccines against HPV infection [33,34,35,36,37,38,39,40,41,42,43,44,45,46,47,48,49,50].

Vaccine	Type	Reference	Study Design	Period	Country	Population	No. Doses	Follow-Up Duration	Clearance Results
VGX 3100	2 synthetic DNA plasmids encoding E6/E7 of HPV16 and HPV18	[33]	Phase IIb	2011–2013	USA Estonia South Africa India Canada Australia Georgia	169 women CIN2/3 VGX-3100 (*n* = 125) or placebo (*n* = 42).	3 doses (weeks 0, 4, 12) + electroporation	36 weeks	49.5% of 107 VGX-3100 recipients and 30.6% of placebo had histopathological regression (*p* = 0.03). Among those with histopathological regression, viral clearance was: - VGX-3100 recipients (78%) - Placebo recipients (57%)
GX-188E	Plasmid encoding fusion protein of HPV 16/18 E6/E7 linked to Flt3L and tpa	[34]	Phase I	2012	Republic of Korea	9 women CIN3	3 doses (weeks 0, 4, 12) + electroporation	36 weeks	Week 12: - viral clearance (4/9 patients) - cytological recovery (3/9 patients) Week 20: - cleared cervical lesions (6/7 patients)-showed durable immunity - high polyfunctional HPV-specific CD8 T-cell response
		[35]	Phase II	2014–2016	Republic of Korea	87 women CIN3 HPV16/18	3 doses (weeks 0, 4, 12) + electroporation	36 weeks	52% of patients at 20 weeks and 67% at 36 weeks presented histopathologic regression; 77% of those with histologic regression showed HPV clearance. HPV clearance and histopathologic association: 20 weeks visit (OR = 13.9; 95% CI, 4.1–47.2) 36 weeks visit (OR = 25.3; 95% CI, 4.8–14.9)
MVA E2	Modified Vaccinia virus of Ankara (MVA) containing bovine PV E2 protein	[36]	Phase I/II	2002–2003	Mexico	78 CIN1/2/3 MVA E2 (*n* = 36) Cryosurgery (*n* = 42)	6 doses (intralesional weekly)	24 weeks	34/36 patients showed complete elimination of precancerous lesions after vaccination. Among patients, 50% completely eliminated HPV, and in remaining 50% of patients, HPV DNA was only 10% of original viral load.
		[37]	Phase II	2002–2004	Mexico	52 women CIN3 2 women CIN2 (34 vaccine/20 control)	6 doses (intralesional weekly)	24 weeks	Histological analysis showed total elimination of high-grade lesions in 20 out of 34 patients, and 11 had a 50% reduction in lesion size. DNA viral load was significantly reduced in MVA E2-treated patients: 12/34 efficiently eliminated all HPV DNA. In some patients, viral load diminished by 95%. In remaining patients, viral load was reduced between 15 and 50%. None of 20 control patients treated by conization eliminated HPV.
		[38]	Phase III	2007–2012	Mexico	1176 women and 180 men with intraepithelial lesions (CIN1/2/3)	Women: 6 doses (intralesional weekly) Men: 5 doses (intraurethral)	24 weeks	89.3% of women and 100% of men achieved complete elimination of lesions, and 2.4% of women showed a reduction in CIN1 HPV DNA clearance: 83%
GTL 001 (ProCervix)	Recombinant E7-CyaA fusion proteins expressed in and purified from *Escherichia* *coli*	[39]	Phase I	2010–2012	Belgium France	47 women HPV16/18^+^ with normal cervical cytology	2 (intradermal 6 weeks apart)	24 weeks	In most groups treated with GTL001 600 mg + imiquimod, mean viral loads decreased over time. In randomized portion of trial, sustained clearance occurred in 5 of 6 (83%) patients treated with highest dose of GTL001 (600 mg) versus 1 of 3 (33%) treated with placebo. Trial not designed to detect significant differences in viral clearance.
		[40]	Phase II	2013–2015	7 countries in Europe	233 patients HPV16/18 with normal or abnormal (ASCUS/LSIL) cytology	2 (intradermal 6 weeks apart)	2 years	No statistical difference in viral clearance between treatment and placebo groups at any point over 2 years
TG4001	MVA encoding IL-2 and modified forms of HPV 16 E6 and E7 proteins	[41]	Phase II	2004–2005	France	21 patients with HPV 16-related CIN2/3	3 doses (1 week interval)	12 months	7/10 clinical responders concomitantly had no HPV 16 mRNA detected (3 other results not available). 8/10 had no HPV 16 DNA present. Median times to disappearance of HSIL: 5 weeks (95% CI, 9–27), Clearance of HPV 16 E6/E7 transcripts: 13.3 weeks (95% CI, 9–18), HR-HPV DNA clearance: 26 weeks (95% CI, 19–33)
Tipapkinogen Sovacivec (TA) therapeutic HPV vaccine	Attenuated vaccinia virus (Wyeth strain) encoding IL-2 and modified forms of HPV 16 E6 and E7 proteins	[42]	Phase IIb	2009–2011	USA Spain Belgium France Finland	206 women >=18 years old with confirmed CIN2/3	3 doses (1 week interval)	30 months	Complete resolution significantly higher in vaccine group for CIN 2/3 regardless of 13 HR-HPV types assayed (24% vs. 10%, *p* < 0.05); as well as for only CIN3, regardless of HR-HPV type (21% vs. 0%, *p* < 0.01). Irrespective of baseline HPV infection, viral DNA clearance was higher in vaccine group compared to placebo (*p* < 0.01). Viral DNA clearance of all CIN2/3 and within baseline CIN3, regardless of HR-HPV type, significantly greater in vaccine group (*p* ≤ 0.01). For specific HR-HPV types, vaccine was superior to placebo for: (1) HPV 16 monoinfected women, (2) any HR-HPV infection except HPV 16, (3) HPV16 co-infected with any other HR-HPV infection.
PepCan	4 peptides HPV16 E6 and *Candida* skin test reagent as novel adjuvant	[43]	Phase I	2007–2010	USA	24 women CIN2/3	4 doses (3 week interval)	12 weeks	HPV 16 became undetectable in 3/16 subjects after vaccination; viral loads significantly decreased in 9 of 16 HPV16-infected women prior to vaccination. Rate of at least one HPV type becoming undetectable at exit was highest (85%) at 50 μg dose. Systemic T-helper type 1 (Th1) cells were significantly increased after vaccination.
		[44]	Phase I	2012–2014	USA	37 women CIN2/3			
Vvax001	Replication-deficient recombinant Semliki Forest virus (rSFV) particles encoding E6 and E7 of HPV16	[45]	Phase II	2021–2023	The Netherlands	18 patients HPV16-positive CIN3	3 doses (3 week interval)	30 months	10/16 (63%) patients exhibited HPV16 clearance 19 weeks after last vaccination (V8). All patients (100%) with histopathologic regression (8/18) exhibited HPV16 clearance at V8. Among non-responders (8/18), two patients exhibited HPV16 clearance at V8, but two other HPV types (HPV52 and HPV31) were detected in their post-treatment biopsies.
ZYC101a	Plasmid-DNA–encoding fragments derived from HPV 16/18 E6/E7	[46]	Phase II	2000–2001	USA Europe	161 women CIN2/3	3 doses (intramuscular)	6 months	Proportion of subjects who resolved tended to be higher after vaccination compared to placebo (43% vs. 27% *p* = 0.12). Subjects younger than 25 years with HPV16 or HPV18 at entry, and subjects with other HPV subtypes at entry, experienced higher rates of resolution than placebo subjects (64% vs. 22% and 73% vs. 25%, respectively)
HspE7 (SGN-00101)	*M. bovis* BCG heat shock protein (Hsp65), linked to HPV 16 E7 protein.	[47]	Phase II	2003–2005	New York, USA	58 women CIN3	3 doses (monthly subcutaneous vaccinations)	8 weeks	22.5% complete pathologic response and 55% partial response. Women who had previous LEEP or ablation for CIN were 2.7 times more likely to have complete response. Decline in HPV dot blot intensity and eventual loss of HPV16 in two patients with complete pathologic response
		[48]	Phase II	2001–2004	California, USA	21 women CIN2/3	4 doses	12 months	35% of women had complete regression of their intraepithelial neoplasia, 5% showed regression to CIN1, 55% had stable disease and one progressed. Regression was correlated with immune response. Viral resolution occurred in only one woman participating in this study.
CIGB-228	Peptide-based HLA-restricted HPV16 E7 epitope adjuvated with VSSP	[49]	Phase I	2007–2008	Cuba	7 women, HLA-A2 positive, CIN2/3 and HPV16 positive.	4 doses (weekly)	12 months	5/7 women had complete and partial regression. T-cell response was induced in all patients. Concomitant clearance of HPV16 from original lesion sites was observed in three patients who had complete response
IGMKK16E7	HPV16 E7-expressing *Lactobacillus casei*-based oral vaccine	[50]	Phase I/II	2019–2021	Japan	165 women CIN-2/3	4 doses (orally at weeks 1, 2, 4, and 8)	16–24 weeks	Histopathological regression to normal (complete response) occurred in 32% of high-dose recipients and 12 placebo. Further improvement of IGMKK16E7 is desired to achieve therapeutic effect on viral clearance.

LEEP—Loop Electrosurgical Excision Procedure (LEEP) or cone biopsy of the cervix.

## Data Availability

No new data were created or analyzed in this study.
